# The role of PKN1 in glioma pathogenesis and the antiglioma effect of raloxifene targeting PKN1

**DOI:** 10.1111/jcmm.17860

**Published:** 2023-07-21

**Authors:** Yubing Hao, Zelin Li, Anling Zhang, Li Sun, Guangxiu Wang, Hu Wang, Zhifan Jia

**Affiliations:** ^1^ Department of Neurosurgery Tianjin Medical University General Hospital. Tianjin Neurological Institute, Laboratory of Neuro‐Oncology, Key Laboratory of Post‐Trauma Neuro‐Repair and Regeneration in Central Nervous System, Ministry of Education. Tianjin Key Laboratory of Injuries, Variations and Regeneration of Nervous System Tianjin P. R. China; ^2^ Clinical College of Neurology Neurosurgery and Neurorehabilitation Tianjin Medical University Tianjin P. R. China; ^3^ Laboratory of Neuro‐Chemistry Tianjin Neurological Institute, Tianjin Medical University General Hospital Tianjin China; ^4^ Department of Neurosurgery Tianjin Huanhu Hospital Tianjin China

**Keywords:** GBM, PKN1, raloxifene, TMZ, YAP

## Abstract

PKN1 (protein kinase N1), a serine/threonine protein kinase family member, is associated with various cancers. However, the role of PKN1 in gliomas has rarely been studied. We suggest that PKN1 expression in glioma specimens is considerably upregulated and positively correlates with the histopathological grading of gliomas. Knocking down PKN1 expression in glioblastoma (GBM) cells inhibits GBM cell proliferation, invasion and migration and promotes apoptosis. In addition, yes‐associated protein (YAP) expression, an essential effector of the Hippo pathway contributing to the oncogenic role of gliomagenesis, was also downregulated. In contrast, PKN1 upregulation enhances the malignant characteristics of GBM cells and simultaneously upregulates YAP expression. Therefore, PKN1 is a promising therapeutic target for gliomas. Raloxifene (Ralo), a commonly used selective oestrogen‐receptor modulator to treat osteoporosis in postmenopausal women, was predicted to target PKN1 according to the bioinformatics team from the School of Mathematics, Tianjin Nankai University. We showed that Ralo effectively targets PKN1, inhibits GBM cells proliferation and migration and sensitizes GBM cells to the major chemotherapeutic drug, Temozolomide. Ralo also reverses the effect of PKN1 on YAP activation. Thus, we confirm that PKN1 contributes to the pathogenesis of gliomas and may be a potential target for Ralo adjuvant glioma therapy.

## 1 INTRODUCTION

Glioma is the most common primary brain tumour, especially its subtype, glioblastoma multiforme (GBM), a highly malignant tumour with a very poor prognosis frequently seen in adults. Glioma development is associated with many genetic and molecular aberrations and substantially changes major signalling pathways. The standard of care for glioma has improved greatly; however, the efficacy is still unsatisfactory,^1^ and the median survival of GBM after diagnosis is only 12–15 months.^2^, ^3^ Therefore, the primary focus of glioma research is to elucidate glioma pathogenesis further and provide new clinical treatment approaches.

Protein kinase N1 (PKN1) belongs to the PKN family. PKN1 is a serine/threonine protein kinase with a catalytic domain homologous to protein kinase C (PKC) and has a regulatory region widely distributed in various tissues, including the brain.^4^ PKN1 is implicated in the progression of prostate, bladder, liver and pancreatic cancer.^5^, ^6^, ^7^, ^8^ However, the expression and the role of PKN1 in glioma have not been elucidated.

The Hippo pathway is involved in tumorigenesis, and YAP (yes‐associated protein) is an essential member of this pathway. YAP translocates and binds to the cell nucleus. Through transcriptional binding partners, YAP promotes glioma cell proliferation, migration and invasion.^9^, ^10^ However, the effect of PKN1 on YAP has not been reported.

Tamoxifen (Tamo), a first‐generation selective oestrogen receptor modulator (SERM), is a chemotherapeutic drug to treat oestrogen receptor positive breast cancer. Tamo inhibits glioma cell proliferation, promotes apoptosis,^11^, ^12^ and increases phototherapy sensitivity in U251 and U87 cell lines.^13^ Tamo induces apoptosis in Temozolomide (TMZ)‐resistant glioma cell lines.^14^ Although Tamo can improve the quality of life and performance status of patients,^15^, ^16^ its effects on time to progression and median survival time of patients with high‐grade glioma are unsatisfactory. We used VIOD,^17^ a bioinformatics service platform developed by the School of Mathematics, Tianjin Nankai University, to predict and analyse the potential drug targeting of PKN1 and found that Ralo, a second generation SERM,^18^ might be directly used against PKN1. Ralo can prevent breast cancer and has fewer side effects than Tamo,^19^, ^20^ and reportedly inhibits prostate cancer, lung cancer, pituitary adenoma and acute lymphocytic leukaemia progression.^21^, ^22^, ^23^, ^24^ In addition, Ralo enhances the chemosensitivity of glioma cells to TMZ in vitro.^25^ However, the therapeutic effect of Ralo on GBM is largely unknown.^26^ In this study, the inhibitory effects of Ralo on GBM cells and its potential targeted protein, PKN1, were investigated, and the effect of Ralo sensitizing GBM cells to TMZ was further identified.

## 2 METHODS AND MATERIALS

### 2.1 Reagents and consumables

Dulbecco's modified Eagle's medium (DMEM), fetal bovine serum (FBS) and Tyrisin (0.25% EDTA) were purchased from Gibco Life Technologies (Grand land, New York, USA). Protease inhibitor, RIPA lysis buffer, dimethylsulfoxide (DMSO), antibiotics (penicillin‐ streptomycin‐gentamicin solution), SDS‐PAGE loading buffer and Trizol were supplied by Solarbio Life Science (Beijing, China). Minute TM cytoplasmic and nuclear extraction kit for separating cytoplasmic and nuclear proteins was purchased from Invent Biotechnologies, Inc (Beijing, China). Antibodies for YAP1, MMP2, Bcl2, PCNA, and GAPDH were purchased from ABclonal Biotech Co. Ltd (Boston, USA) and PKN1 was supplied by Abcam (Cambridge, UK). Goat anti‐mouse, anti‐rabbit and immunohistochemical (IHC) kits were supplied by ZSGB‐Bio Ltd (Beijing, China). Polyvinylidene fluoride membranes were purchased from Millipore (Bedford, MA, USA). Matrigel was purchased from Becton, Dickinson and Company (NY, USA). Transwell chambers (8‐μm pore size) were purchased from Corning Inc (Shanghai, China) for Transwell assays. Cell counting kit‐8 (CCK‐8) was purchased from Dojindo Molecular Technologies, Inc. (Kumamoto, Japan). Serum‐free cell cyto‐preservation was purchased from NCM Biotech Co. Ltd (Suzhou, China). The apoptosis detection kit, Annexin‐V FITC and propidium iodide (FITC/PI) were purchased from Absin Bioscience Inc. (Shanghai, China). The transfection reagent of EntransterTM‐R4000 was purchased from Engreen Biosystem, Ltd (Beijing, China).

Tissues chips of glioma specimens were supplied by Alena Biotechnology (Shanxi, China, Art. No: GL803C H060), including three cases of WHO I, 26 of WHO II, four of WHO III, 38 of WHO IV, five of non‐tumour brain tissue and five dots on the chip were unidentified.

Small interfering RNA of PKN1 (siR‐PKN1, 5′ to 3′: AAG GGC ACG GGA ACT GGA GTT; siR‐NC (negative control): UUC UCC GAA CGU GUC ACG UTT; ACG UGA CAC GUU CGG AGA ATT) and PCR primers (PKN1 forward: GAG GTG GAG AGC CTG ATG TG; reverse: CTG GAA ACA GCC GAA GAG GT; YAP forward: GCG GAA TAT CAA TCC CAG CAC; reverse: GGT GCC ACT GTT AAG GAA AGG) were purchased from GenePharma (Shanghai, China). PKN1 recombinant adenoviruses (ADV‐PKN1) were purchased from Vigene Biosciences (Shandong, China). Ralo hydrochloride (C28H28CINO4S) and TMZ (C6H6N6O2) were purchased from TargetMol Chemicals Inc (Boston, MA USA).

### 2.2 Glioma cell lines and cell culture

The human A172, U87, and LN229 GBM cell lines were purchased from Zhong Qiao Xin Zhou Biotechnology Co. Ltd (Shanghai, China). The LN18, U118, U251, LN308 and SNB19 GBM cell lines were obtained from the Neuro‐Oncology Laboratory, Tianjin Institute of Neurology. All cell lines were cultured in DMEM containing 10% FBS and in a 37°C 5% CO_2_ incubator and subcultured every 2–3 days.

### 2.3 Peritumor and glioma brain tissues extraction and analysis

A total of 37 glioma specimens, including 14 Grade I–II, 12 Grade III, 11 Grade IV, and 10 nontumorous brain tissues (NB tissues), were obtained from the Department of Neurosurgery, Tianjin Medical University General Hospital from 2015 to 2016. Tissue specimens and clinical information were obtained according to the regulations and internal bio‐safety and bioethics guidelines of the Tianjin Medical University General Hospital Ethical Committee. Although the current WHO classification of central nervous system tumours has progressed from using only histopathological findings to incorporate molecular alterations, since all our glioma samples were collected before 2016, the histopathological diagnosis of glioma tissues could only be independently classified according to the typical histopathological findings by three neuropathologists.

The explant tissues were ground into a powder in liquid nitrogen, and 500 ul RIPA buffer was added. The tissues were then ultrasonically decomposed for 1 min, incubated on ice for 40 min, and centrifuged at 12000 rpm for 20 min. The supernatant was obtained, and appropriate 4× SDS‐PAGE loading buffer was added, and boiled in a water bath for 5 min. The tissues were stored at −80°C for western blotting.

### 2.4 IHC detection of tissue chip

The tissue chip was incubated overnight with the appropriate primary antibody (PKN1, 1:50 dilution) in a 4°C wet box. The chip was then incubated with a secondary antibody and stained using 3,3′‐diaminobenzidine and haematoxylin. Dehydration and sealing of the chip followed, and the chip was scanned using a tissue chip scanner (Pannoramic MIDI, 3D HISTECH) and a semi‐quantitative analysis with histochemistry score (H‐SCORE) was performed.

### 2.5 Cell transfection and protein detection

The siR‐PKN1 was transfected into A172 and U87 cells using an EntransterTM‐R4000 when the cultured cell density reached 70–80%, and ADV‐PKN1 was transfected into LN229 cells using ADV helper reagent according to the manufacturer's instructions. Proteins were extracted 48 h after transfection using RIPA lysis buffer (PMSF, 1:100), and cytoplasmic and nuclear proteins were extracted using Minute TM cytoplasmic and nuclear extraction kits. Western blotting detected PKN1, MMP2, Bcl2, PCNA, YAP nuclei and cytoplasm distribution.

Ralo was dissolved in DMSO to a concentration of 50 mM. The 50% and 25% inhibition concentration (IC50 and IC25, respectively) values of Ralo in A172 and U87 cells were measured using the CCK‐8 assay. A172 and U87 cells were then treated with corresponding concentrations of IC50 and IC25, and the proteins were extracted. PKN1, MMP2, Bcl2 and PCNA expression, and YAP expression in the cytoplasm and nuclei were analysed through western blotting.

### 2.6 Glioma cell line proliferation analysis and colony‐forming assay

Glioma cells were inoculated into 6‐well plates, 500 cells/well, and cultured for 14 days. Cells were fixed with 4% paraformaldehyde for 10 min, then stained with 0.4% crystal violet for 10 min, and colony dots were photographed and counted.

For the CCK‐8 assay, glioma cells were inoculated into 96‐well plates and proliferation capacity was detected using the CCK‐8 kit according to the manufacturer's instructions. Results are represented as the relative survival rate compared to the control.

### 2.7 Real‐time quantitative PCR to detect PKN1 and YAP expression

The total RNA was extracted from glioma cells using Trizol. RNA was reverse transcribed, and real‐time quantitative PCR was conducted on a Bio‐Rad Cycler system using the SYBER Premix DimerEraser. Fold changes were calculated by relative quantification. Data are shown as fold change (2‐ΔΔCT method) and analysed initially using Opticon Monitor Analysis Software V 2.02 software (MJ Research).

### 2.8 Flow cytometry to detect cell apoptosis

Cells of parental and positive groups in the logarithmic growth period were collected, centrifuged and resuspended in a binding buffer containing FITC/PI. Apoptosis was detected and analysed using FACSCalibur (Becton, Dickinson and Company) and FlowJo software (FlowJo LLC).

### 2.9 Glioma cell invasion and migration

The bottom of the Transwell chambers was coated with diluted Matrigel (Matrigel:DMEM = 1:4). Parental and positive group cells were resuspended using serum‐free DMEM. The DMEM (200 ul) was added to the upper chamber containing 1 × 10^4^ cells, whereas 500 ul DMEM with 10% FBS was added to the lower chamber as a chemo‐attractant. After 24 h of incubation, the chambers and substrate were removed. The invading cells were fixed with 4% paraformaldehyde for 10 min and stained with 0.4% crystal violet for 10 min. The invading cells were determined in at least three fields from each chamber using inverted light microscopy.

For the migration assay, 2 × 10^5^ parental and positive cells were seeded on six‐well plates and cultured. When the cell density reached 80–90%, “+” graph scratch wounds were made through the center of the plate using a 200 μL pipette tip, and cells were cultured. At least three random visual fields were selected to be observed and photographed using an inverted light microscope at 0, 12 and 24 h. The experiment was repeated thrice.

### 2.10 Statistical analysis

Statistical analysis was performed using SPSS Statistics 17.0 (SPSS, Chicago, IL, USA) of anova analysis or Student's test. *p < 0.05 was considered statistically significant. The data are presented as the means±standard deviation, and the graphs were drawn using GraphPad Prism 6.0 (GraphPad Software).

## 3 RESULTS

### 3.1 PKN1 expression in GBM cell lines and glioma tissues

**FIGURE 1:**
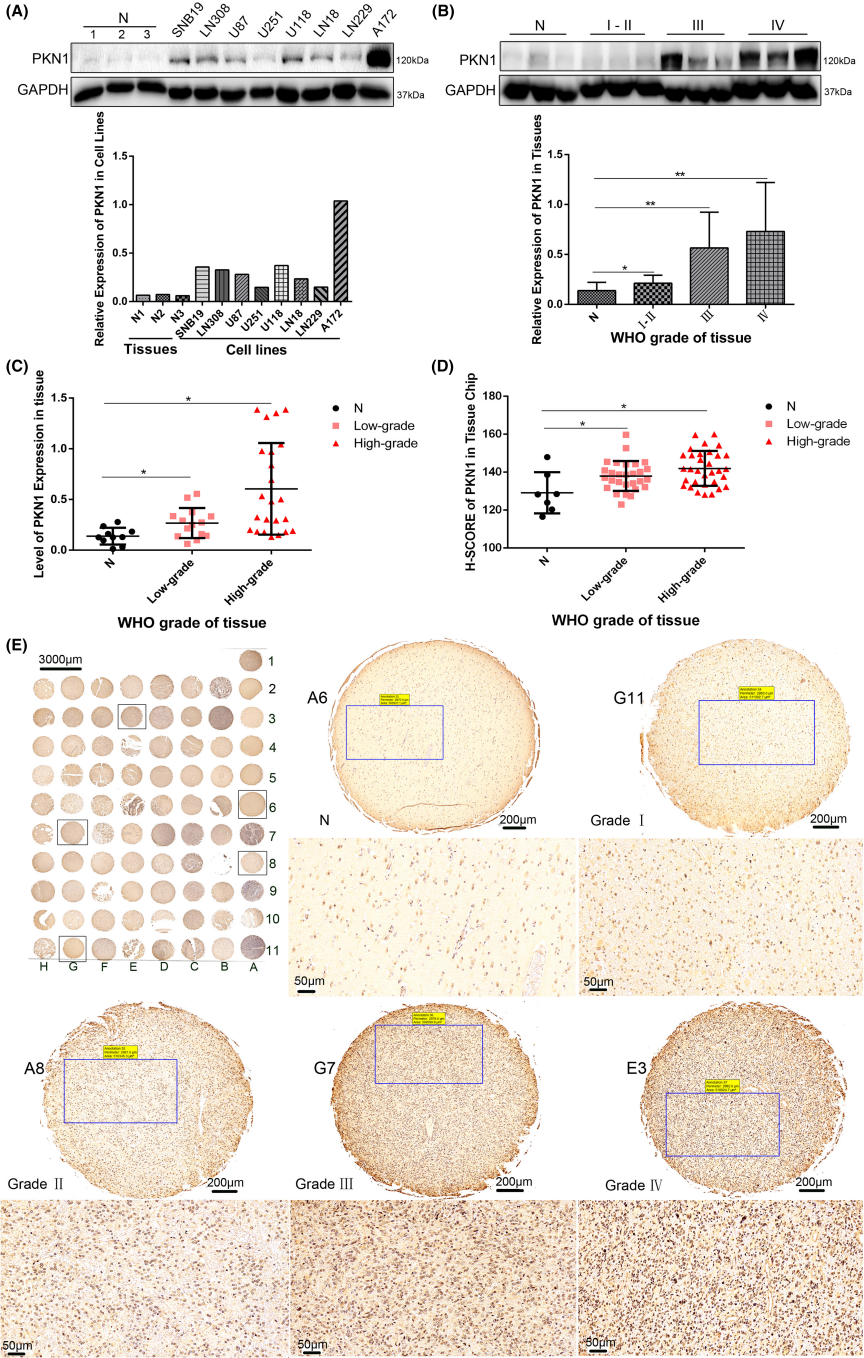
PKN1 expression in GBM cell lines and glioma tissues (*p < 0.05, **p < 0.01; compared with NB tissues) (A) The protein expression level of PKN1 in GBM cell lines (A172, U87, LN229, LN18, U118, U251, LN308, and SNB19) was detected using western blotting, and the protein rations for all lanes were included in western blots; the NB tissues served as a negative control. (B) The protein expression level of PKN1 in 37 glioma specimens and 10 NB tissues was detected using western blotting, and the protein rations for all lanes were included in western blots. (C) The relative grey value of protein expression of PKN1 in NB and glioma tissues. (D): The H‐SCORE of PKN1 of each tissue dot in the tissue chip. (E): Immunohistochemistry was used to detect PKN1 expression in the brain tissue microarray. 400×, scale bar 50 μm. N: A2, A3, A4, A5, A6; low‐grade (GradeI: G10, G11, H10; Grade II: A8–10, B4–11, C2–6, G9, H2–9, H11); high‐grade (Grade III: G2–4, G5–8; Grade IV: C7–11, D2–11, E2–11, F2–8, F9–11); A1, A7, A11, B2 and B3 are the null‐tissue points.

The proteins of eight GBM cell lines (A172, U87, LN229, LN18, U118, U251, LN308 and SNB19) and three NB tissues were collected. PKN1 expression in GBM cell lines was higher than that in NB tissues. PKN1 expression in A172 cells was the highest, LN229 cells were the lowest and U87 cells were medium (Figure 1A).

We detected the PKN1 protein in 37 glioma specimens (Grade I–II: *n* = 14, Grade III: *n* = 12, and Grade IV: *n* = 11,) and in 10 NB tissues. Therefore, PKN1 expression was upregulated in glioma specimens compared with NB tissues and positively correlated with tumour grades (Figure 1B,C). PKN1 expression in high‐grade (WHO III/IV) glioma was significantly higher than that in low‐grade glioma (WHO I/II) (Figure 1B,C).

PKN1 expression was detected in NB tissues and glioma specimens. PKN1 expression was more upregulated in gliomas than in NB tissues, and the immunopositive rate and staining intensity were positively correlated with glioma grade. Furthermore, PKN1 was only detected in the cytoplasm in NB tissues. Simultaneously, PKN1 was presented in the cytoplasm and nucleus with increasing tumour grade. The semi‐quantitative scores of low‐ and high‐grade glioma tissues were higher than in the control group, and the higher the glioma grade, the higher the H‐SCORE (Figure 1D,E). These findings indicated that the PKN1 expression level was positively correlated with the tumour grade.

### 3.2 PKN1 plays a vital role in glioma pathogenesis

**FIGURE 2:**
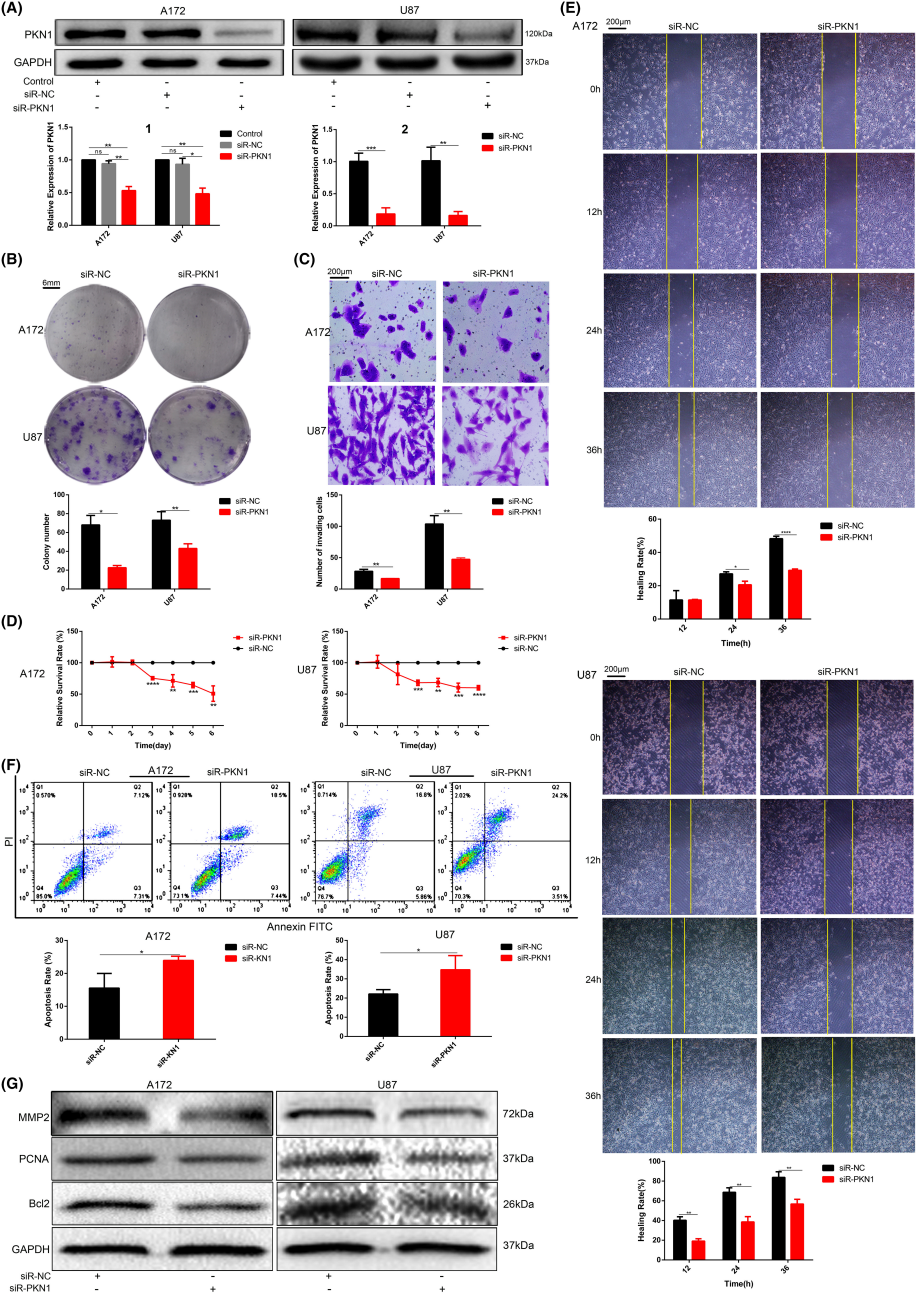
Effect of PKN1 knockdown on the biological behaviours of GBM cells (*p < 0.05, **p < 0.01, ***p < 0.001, ****p < 0.0001; compared with the siR‐NC groups). (A) A172 and U87 GBM cells were transfected with siR‐PKN1 followed by western blotting and RT‐PCR (Figure A2) to detect PKN1 expression. The transfection ratios were analysed (Figure A1). (B) Colony formation assay of A172 and U87 cells transfected with siR‐PKN1, scale bar 6 mm. (C) Transwell assay of A172 and U87 cells transfected with siR‐PKN1, 100×, scale bar 200 μm. (D) The cell proliferation of A172 and U87 cells transfected with siR‐PKN1 was detected using the CCK‐8 assay. (E) The wound healing assay of A172 and U87 cells transfected with siR‐PKN1. 100×, scale bar 200 μm. (F) Apoptosis of A172 and U87 cells transfected with siR‐PKN1 was detected by flow cytometry. (G) MMP2, PCNA, and Bcl2 were expressed in A172 and U87 cells transfected with siR‐PKN1, and the loading control was GAPDH.

**FIGURE 3:**
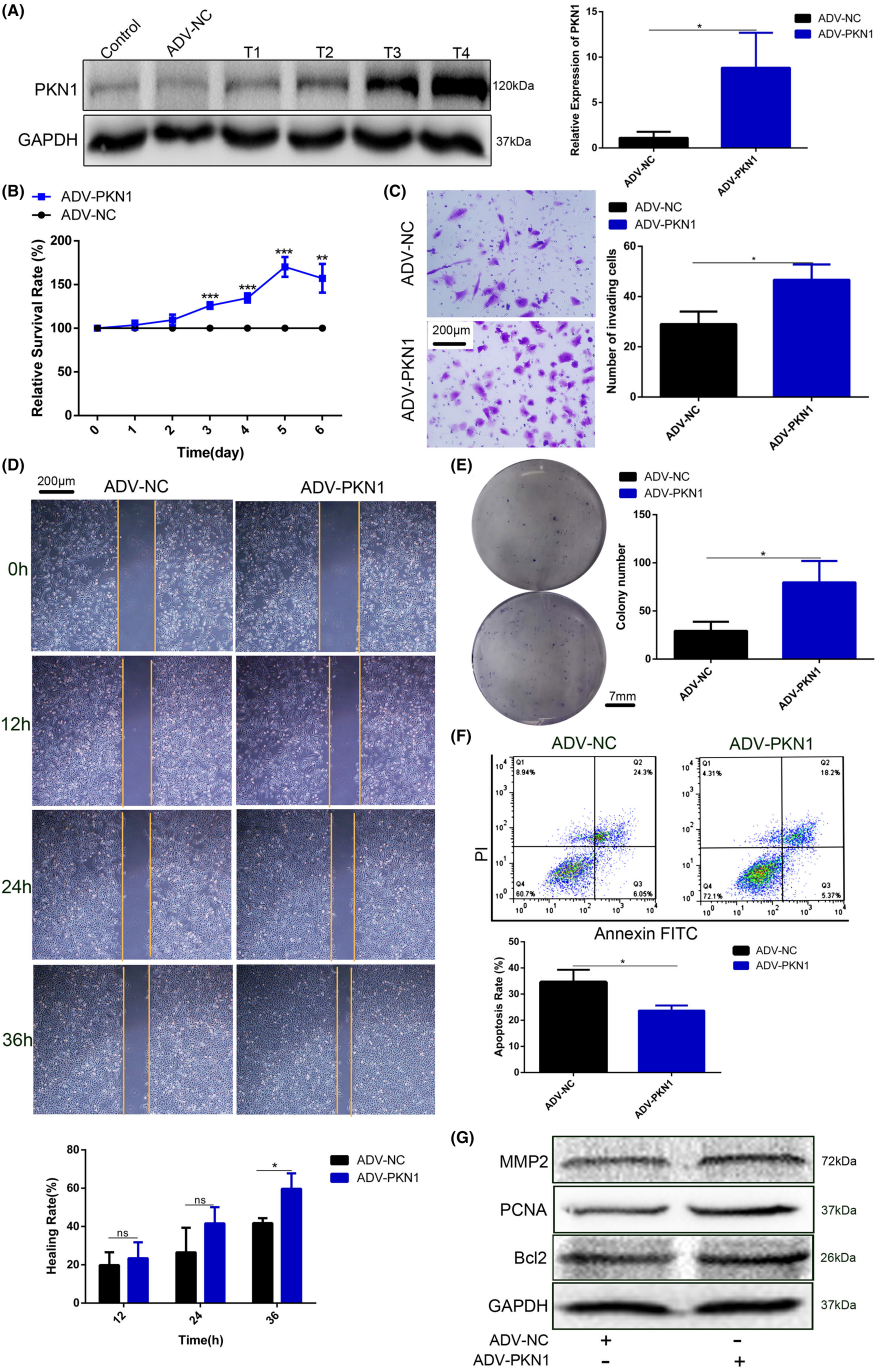
Effect of PKN1 overexpression on the biological behaviours of LN229 cells (*p < 0.05, **p < 0.01, ***p < 0.001; compared with the ADV‐NC group). (A) LN229 cells were transfected with different ADV‐PKN1 concentrations (T1, T2, T3, and T4) followed by western blotting and RT‐PCR analysis to detect the PKN1 expression level; GAPDH was the loading control. (B) The CCK‐8 assay examined the LN229 cell proliferation transfected with ADV‐PKN1. (C) The invasive ability of LN229 cells transfected with ADV‐PKN1 detected by the Transwell assay, 100×, scale bar 200 μm. (D) The wound healing assay was used to examine the migration of LN229 cells transfected with ADV‐PKN1. 100×, scale bar 200 μm. (E) LN229 cells transfected with ADV‐PKN1 detected by colony formation assay, scale bar 7 mm. (F) Flow cytometry assayed the apoptosis of LN229 cells transfected with ADV‐PKN1. (G) MMP2, PCNA, and Bcl2 expression was examined using western blotting in LN229 cells transfected with ADV‐PKN1.

When siR‐PKN1 was transfected into A172 and U87 cells with higher PKN1 expression, PKN1 expression was inhibited. In contrast, in LN229 cells, which had low PKN1 expression, when transfected with ADV‐PKN1, PKN1 expression was upregulated. The PKN1 expression level in GBM cell lines was detected using western blotting and real‐time PCR (Figures 2A and 3A). Four different ADV‐PKN1 concentrations (T1–T4) were tested; T4 appeared to be the most effective and was identified as a working concentration in the follow‐up studies (Figure 3A).

The proliferation viability of the siR‐PKN1 groups significantly decreased compared to the siR‐NC groups (Figure 2B,D), whereas the GBM cells proliferation in the ADV‐PKN1 group was enhanced (Figure 3B,E).

In the wound healing assay, the migration ability of PKN1 knockdown cells was significantly reduced, and PKN1 overexpression drastically increased the mobility of GBM cells (Figures 2E and 3D) compared with that in the control group. The number of invading cells in A172 and U87 cells transfected with siR‐PKN1 was significantly decreased, whereas the invasion of LN229 cells transfected with ADV‐PKN1 was increased compared to the control group (Figures 2C and 3C).

Moreover, the apoptosis of A172 and U87 cells in the siR‐PKN1 group was increased compared with the control cell group, indicating that PKN1 knockdown induced apoptosis, whereas PKN1 overexpression suppressed LN229 cell apoptosis (Figures 2F and 3F).

MMP2, PCNA and Bcl2 expression was significantly decreased in A172 and U87 cells transfected with siR‐PKN1, but their expression was increased in LN229 cells transfected with ADV‐PKN1 (Figure 2G and 3G). These results coincided with the effect of PKN1 on cell proliferation, invasion and apoptosis, as described above.

PKN1 expression was upregulated in gliomas, and its expression was positively correlated with tumour malignancy. Downregulating PKN1 contributed to reduced tumour cell proliferation, invasion, and migration and induced GBM cells apoptosis. However, PKN1 overexpression enhanced tumour cell proliferation, invasion and migration and suppressed apoptosis. These results suggest that PKN1 plays an essential role in GBM pathogenesis.

### 3.3 The effects of Ralo targeting PKN1 on the biological behaviour of GBM cells

The IC50 value, the drug concentration required for 50% cell growth inhibition, of Ralo for A172 and U87 cells were 34 μM and 32 μM, respectively (Figure 4‐1A). The IC25 value, the drug concentration required for 25% cell growth inhibition, of Ralo for A172 and U87 cells were 25 μM and 15 μM, respectively (Figure 4‐1A). Therefore, 34 μM and 25 μM Ralo were administered to A172 cells, whereas 32 μM and 15 μM Ralo were administered to U87 cells to observe the effect of Ralo on PKN1 expression and proliferation, invasion, and apoptosis of GBM cells.

**FIGURE 4‐1:**
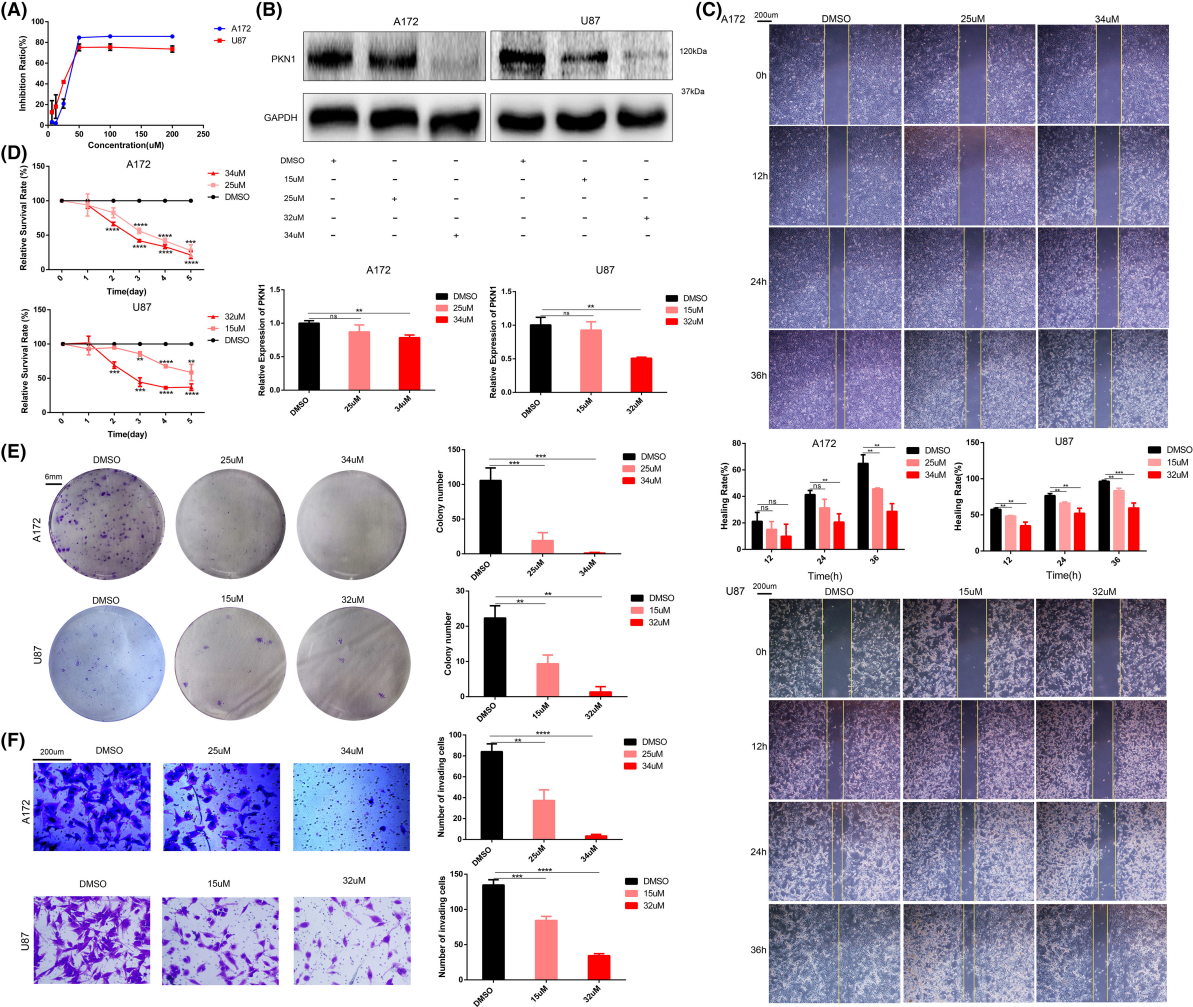
Effect of Ralo on the biological behaviours of GBM cells (*p < 0.05, **p < 0.01, ***p < 0.001, ****p < 0.0001; compared with the DMSO group, ^#^ *p* < 0.05, ^##^ *p* < 0.01, ^###^ *p* < 0.001; compared with the ADV‐NC‐DMSO group). (A) The CCK‐8 assay detected the IC50 and IC25 values of Ralo in A172 and U87 cells. (B) Western blotting and RT‐PCR were used to detect PKN1 expression in GBM cells treated with different Ralo concentrations. (C) The migration ability of GBM cells detected using the wound healing assay, 100×, scale bar 200 μm. (D) Proliferation of A172 and U87 cells treated with Ralo was detected using the CCK‐8 assay. (E) Colony formation assay of GBM cells treated with Ralo, scale bar 6 mm. (F) Transwell assay of GBM cells, 100×, scale bar 200 μm.

PKN1 expression in A172 and U87 cells was significantly reduced (Figure 4‐1B). For Ralo‐treated A172 and U87 cells, the proliferation, invasion, and migration were significantly inhibited (Figure 4‐1C–4‐1F). Furthermore, Ralo treatment also induced apoptosis in GBM cells (Figure 4‐2A). MMP2, PCNA and Bcl2 expressions were correspondingly decreased in the Ralo‐treated cell group, which showed a dose‐dependent manner on GBM cells (Figure 4‐2B).

**FIGURE 4‐2:**
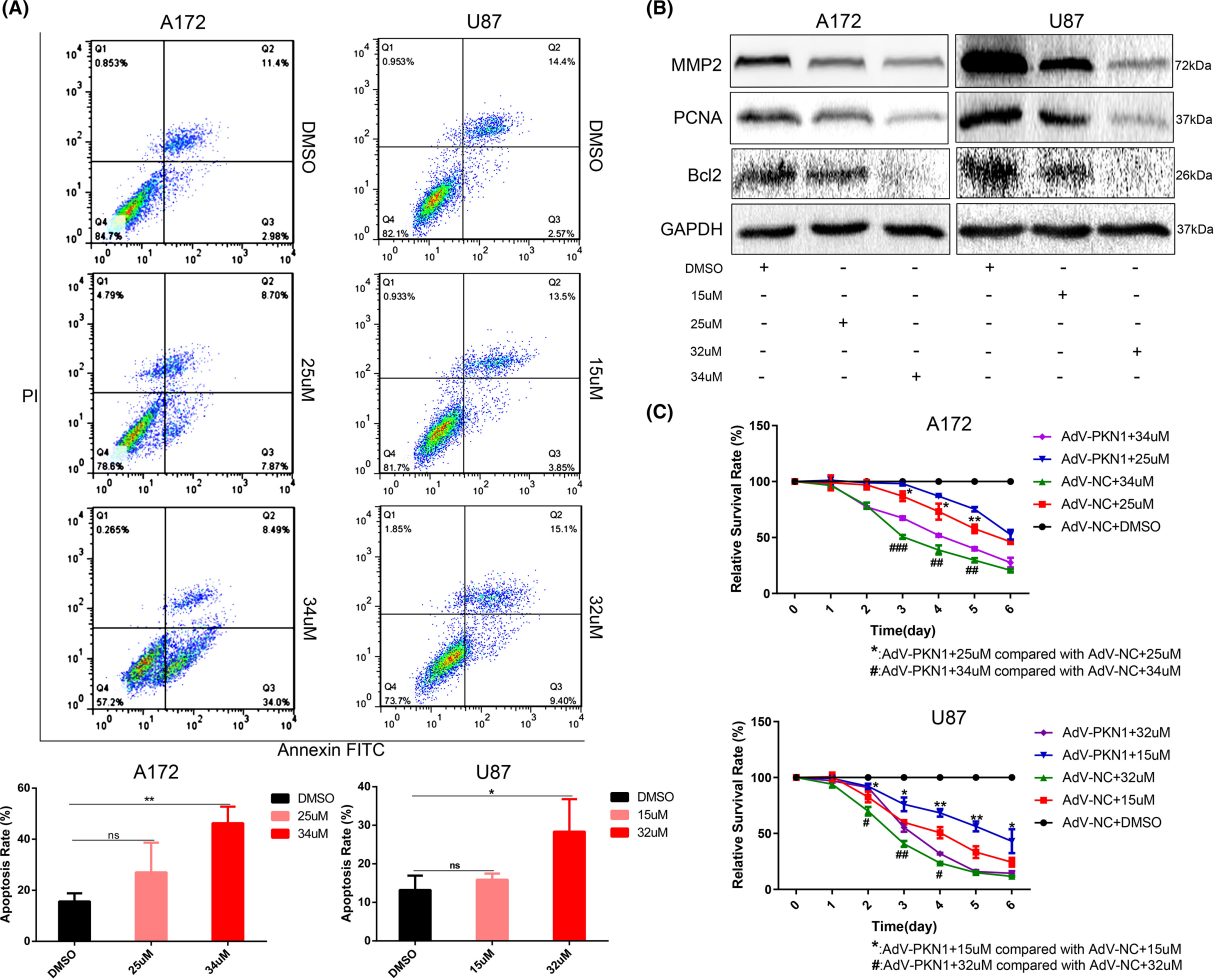
**(**continue Figure 4‐1 **)**. (A) Apoptosis detected via flow cytometry. (B) MMP2, PCNA, and Bcl2 expression in A172 and U87 cells treated with Ralo were examined using western blotting. (C) Proliferation of A172 and U87 cells treated with Ralo alone and ADV‐PKN1 with Ralo.

To further determine if Ralo exerts its effect on GBM cells by targeting PKN1, ADV‐PKN1 was used to rescue the inhibitory effect of Ralo on PKN1. The inhibitory effect of Ralo on GBM cell proliferation was partially blocked by ADV‐PKN1 (Figure 4‐2C). These findings further identified that Ralo partially inhibited GBM cell growth by targeting PKN1.

### 3.4 PKN1 transcriptionally regulates YAP to promote glioma proliferation

**FIGURE 5:**
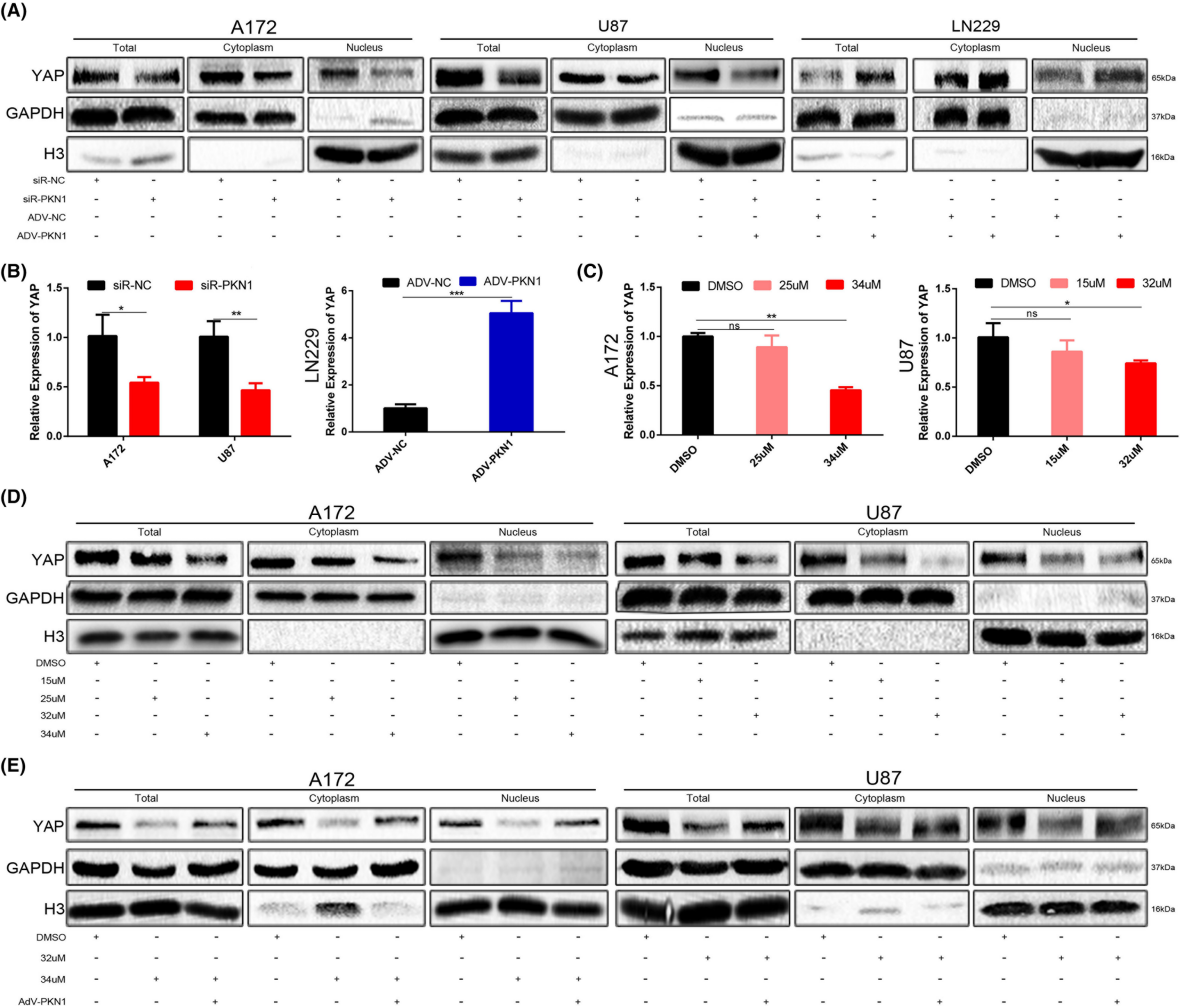
The effect of PKN1 and Ralo treatment on YAP expression in GBM cell lines (*p < 0.05, **p < 0.01; compared with siR‐NC group and DMSO group). (A) After siR‐PKN1 transfection in A172 and U87 cells and ADV‐PKN1 transfection in LN229 cells, the total YAP expression and its distribution in the cytoplasm and nucleus were examined through western blotting. (B) RT‐PCR detected the YAP expression in GBM cells transfected with siR‐PKN1 or ADV‐PKN1. GAPDH was the loading control. (C) RT‐PCR detected the YAP expression in A172 and U87 cells treated with Ralo; GAPDH was the loading control. (D) YAP expression and its distribution in cytoplasm and nucleus after Ralo treatment. (E) ADV‐PKN1 was transfected into Ralo‐treated GBM cells to detect the total YAP expression and its distribution in the cytoplasm and nucleus.

PKN1 regulated YAP expression and activity, a key effector of the Hippo signalling cascade. PKN1 knockdown decreased the YAP mRNA and protein expression. PKN1 downregulation also inhibited the nuclear translocation of YAP, whereas PKN1 overexpression promoted mRNA and protein expression and nuclear translocation of YAP (Figure 5A,B).

In the Ralo treatment groups, YAP mRNA and protein expression significantly decreased, and the nuclear translocation of YAP was also inhibited. The inhibitory effect of Ralo positively correlated with the drug dosage (Figure 5C,D). To confirm whether Ralo suppressed YAP by targeting PKN1, ADV‐PKN1 was added to Ralo‐treated cell groups to restore PKN1 expression. PKN1 upregulation reversed the inhibitory effect of Ralo on YAP expression and nuclear translocation (Figure 5E). These findings indicated that Ralo inhibited YAP expression and activity through PKN1 downregulation.

### 3.5 Ralo enhances TMZ sensitivity by targeting PKN1

**FIGURE 6:**
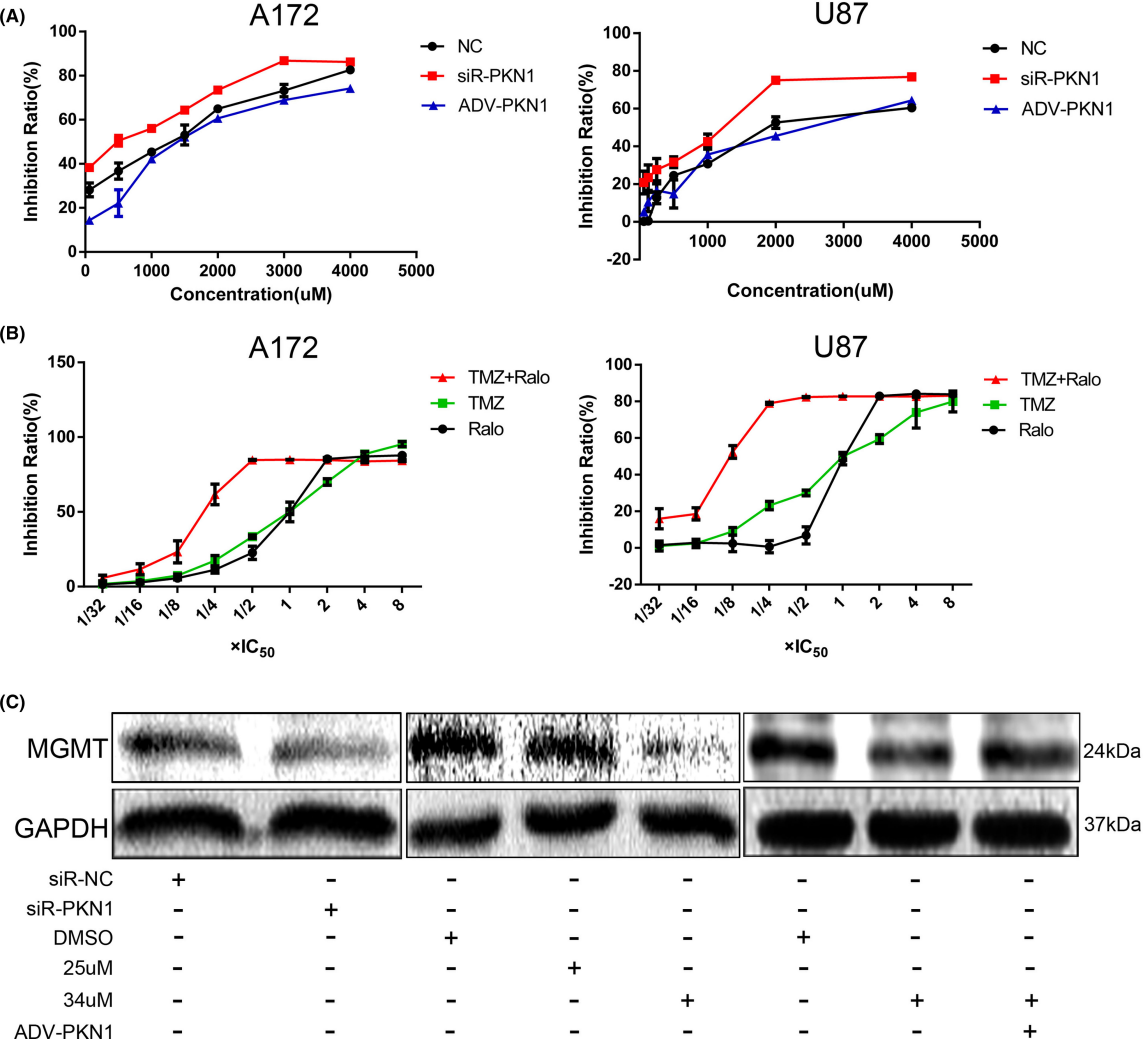
The effect of PKN1 and Ralo on the IC50 value of TMZ and the effect of Ralo on MGMT expression in A172 cells. (A) The effect of PKN1 knockdown and overexpression on the IC50 value of TMZ in A172 and U87 GBM cells. (B) The effect of Ralo and Ralo combined with TMZ on the IC50 value of TMZ and Ralo in A172 and U87 GBM cells. (C) The effect of PKN1 and Ralo treatment on MGMT expression in A172 GBM cells.

The IC50 values of TMZ in the NC, siR‐PKN1, and ADV‐PKN1 groups of A172 cells were 1306.4, 862.3, and 1409.8 μM, respectively, whereas, in U87 cells, the IC50 values were 1877.9, 1226.9 and 2489.9 μM, respectively (Figure 6A). The IC50 value of TMZ in the PKN1 knockdown group was significantly lower than in the control group. The IC50 value in the PKN1 overexpression group was higher than in the control group. Therefore, PKN1 could enhance TMZ resistance in GBM cells.

We administered Ralo combined with TMZ to GBM cells and detected the IC50 value of the two drugs. The results showed that in A172 cells, the IC50 values of Ralo and TMZ were 26.54 μM and 265.5 μM, respectively, whereas, in U87 cells, they were 23.3 μM and 233.01 μM, respectively (Figure 6B). IC50 values of TMZ in A172 cells and U87 cells were 1306.4 μM and 1877.9 μM when TMZ was administered alone, and IC50 of Ralo in A172 cells and U87 cells were 34 μM and 32 μM when Ralo was given alone, as demonstrated above. The IC50 of combined Ralo and TMZ treatment was much lower than single drug treated alone. Using the combined action index (CI) value to identify the therapeutic effect of Ralo combined with TMZ, CI=IC50_R1_/IC50_R2_ + IC50_T1_/IC50_T2_, IC50_R1_, and IC50_T1_ were the IC50 values of Ralo and TMZ for GBM cells, and IC50_R2_ and IC50_T2_ were the IC50 values of Ralo and TMZ singly used for GBM cells. CI values were quantitatively defined as CI = 1 (superposition effect), CI<1 (synergistic effect) and CI>1 (antagonistic effect). The CI values of A172 and U87 cells were 0.95 and 0.85, respectively, indicating that Ralo and TMZ have synergistic effects to a certain degree in glioma treatment (Figure 6B).

Knocking down PKN1 suppressed MGMT expression in glioma cells. Ralo also inhibited MGMT, whereas PKN1 overexpression reversed the inhibitory effect of Ralo on MGMT (Figure 6C), indicating that Ralo enhanced TMZ sensitivity via PKN1.

Therefore, Ralo can exert a synergistic therapeutic effect with TMZ through PKN1.

## 4 DISCUSSION

PKN1 correlates with multiple cancers. In prostate cancer, PKN1 enhances the proliferation and migration of prostate cancer cells by regulating the PKN1‐WDR5‐Kat8 and PKN1‐H3T11P‐WDR5‐H3K4Me3 signalling pathways.^5^, ^27^, ^28^ In pancreatic ductal adenocarcinoma (PDAC), PKN1 acts as a downstream regulator for the FAK/PI3K/AKT signalling pathway, promoting PDAC cell proliferation.^29^ However, the role of PKN1 in glioma has not been reported.

In the present study, PKN1 expression was upregulated in glioma cell lines and specimens. Furthermore, a positive correlation was observed between the PKN1 expression level and histopathological tumour grades. In addition, IHC staining of glioma tissue microarray showed that PKN1 was detected in the cytoplasm of low‐grade specimens^8^; however, PKN1 was detected in the cytoplasm and nucleus in high‐grade gliomas. This finding indicates that PKN1 translocates from the cytoplasm to nuclei in the malignant glioma cell transformation.

To further elucidate the role of PKN1 on glioma pathogenesis, we knocked down PKN1 in GBM cell lines and found that proliferation, migration and colony formation of GBM cells were suppressed. In contrast, apoptosis was induced when PKN1 was knocked down. On the contrary, PKN1 overexpression promoted GBM cell proliferation and invasion and inhibited cell apoptosis. MMP2, PCNA and Bcl2 expression were downregulated in A172 and U87 cells transfected with siR‐PKN1. In contrast, the expressions were upregulated in LN229 cells transfected with ADV‐PKN1, which coincided with the effect of PKN1 on cell proliferation, invasion and apoptosis. These results suggested that PKN1 played an oncogenic role in GBM pathogenesis.

PKN1 was predicted to be the target protein of Ralo by VIOD, the software for predicting the interaction between a given drug molecule and its targeted proteins, developed from an earlier algorithm by the bioinformatics team from the School of Mathematics, Tianjin Nankai University. Ralo regulates oestrogen receptor activity and can effectively prevent and treat breast cancer^19^ and reverse the malignant phenotype of various non‐oestrogen receptor‐dependent tumours, such as pituitary adenoma, acute lymphoblastic leukaemia, prostate cancer and lung cancer.^20^, ^21^, ^22^, ^23^, ^24^ In GBM, Ralo treatment can reportedly achieve additional benefits when combined with TMZ in vitro.^25^

We identified that Ralo suppressed proliferation, invasion, migration and induced apoptosis of GBM cells. These findings indicated that Ralo could affect the biological behaviours of GBM cells and had a significant anti‐tumour effect. Ralo inhibited the expression level of PKN1 in GBM cells dose‐dependently. The inhibitory effect of Ralo on GBM cell proliferation could be reversed by PKN1 transfection. This evidence demonstrated that Ralo could exert its anti‐tumour effect by targeting PKN1 in GBM cells.

The Hippo signalling pathway plays a vital role in tumour progression. The core members of the Hippo pathway, YAP, and TAZ, promote growth, metastasis and resistance to therapy for solid tumours, including GBM.^30^ PKN1 promotes the expression and nuclear translocation of YAP in GBM cells, thus upregulating YAP activity. Ralo treatment significantly inhibited YAP expression and nuclear translocation, whereas PKN1 overexpression reversed the effect of Ralo on YAP expression and bio‐activity. Therefore, PKN1 enhanced YAP expression and activity to exert biological functions in GBM, and Ralo inhibited YAP by suppressing PKN1.

As a tumour with a poor prognosis, the median survival of GBM is less than 1 year. TMZ is the first‐line chemotherapeutic drug in the standard of care for GBM to improve patient survival.^31^ TMZ resistance weakens the TMZ response in GBM^32^; the anti‐tumour effect of TMZ with a sensitizer will be more effective. Searching for drugs that enhance the sensitivity of GBM to chemotherapy is the focus of treatment. In this study, the CI value^33^ of Ralo combined with TMZ was less than 1, indicating that the combination of Ralo and TMZ suppressed GBM cell proliferation more significantly than TMZ alone, and Ralo enhanced the sensitivity of GBM cells to TMZ.

The O‐6‐methylguanine‐DNA methyltransferase (MGMT) is responsible for the repair of the DNA damage induced by TMZ. MGMT repairs O6‐meG lesions by transferring the alkyl group from guanine to a cysteine residue.^34^ MGMT overexpression contributes to TMZ resistance, whereas MGMT downregulation in glioma cells increases the tumour sensitivity to the cytotoxic effects of TMZ. We also identified that Ralo repressed MGMT and increased tumour cell sensitivity to TMZ by inhibiting PKN1.

## 5 CONCLUSIONS

**FIGURE 7:**
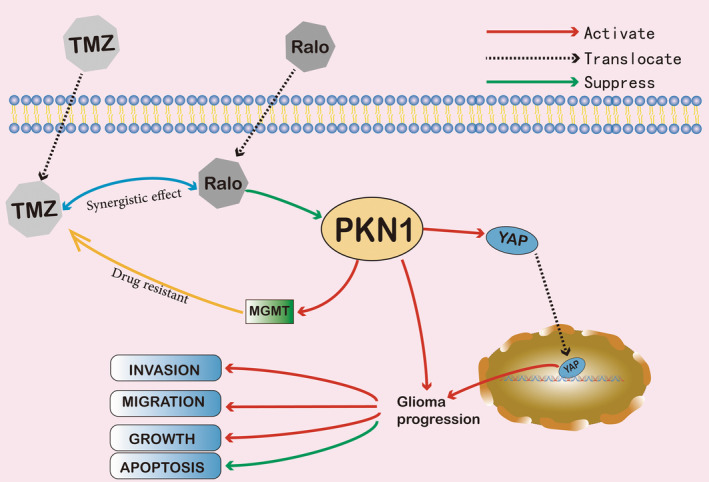
The conceptual model of the PKN1/YAP axis involves the malignant progression of glioma cells. PKN1 promotes the malignant progression of glioma and the anti‐tumour effect of Ralo by targeting PKN1.

We identified that PKN1 is upregualted in glioma, and the promoting role of PKN1/YAP in glioma pathogenesis. Rlao has been proved to inhibit glioma progression by targeting PKN1, and its synergistic effect with TMZ in glioma. These results may offer a new clue for the treatment of GBM (Figure 7).

## AUTHOR CONTRIBUTIONS

**Yubing Hao:** Conceptualization (equal); data curation (lead); formal analysis (equal); project administration (equal); software (equal); supervision (equal); writing – original draft (lead). **zelin li:** Data curation (equal); software (equal); writing – original draft (equal). **Anling Zhang:** Formal analysis (equal); funding acquisition (equal); project administration (equal); resources (equal). **Li Sun:** Funding acquisition (equal); project administration (equal); resources (equal); supervision (equal). **Guangxiu Wang:** Project administration (equal); resources (equal); supervision (equal). **Hu Wang:** Conceptualization (lead); project administration (equal); resources (lead). **Zhifan Jia:** Conceptualization (lead); formal analysis (equal); funding acquisition (lead); project administration (lead); resources (lead); supervision (lead); writing – original draft (lead).

## FUNDING INFORMATION

This work was supported by the National Natural Science Foundation of China (grant no. 30872985, 81,101,915 and 81,571,201). Science and technology project of Tianjin Health Commission (grant no. MS20024).

## CONFLICT OF INTEREST STATEMENT

The authors declare that there is no conflict of interest.

## ETHICS STATEMENT

The Tianjin Medical University General Hospital Ethical Committee approved the study. Tissue samples were obtained from the Department of Neurosurgery, Tianjin Medical University General Hospital. Tissue specimens and clinical information were obtained according to the regulations and internal bio‐safety and bioethics guidelines of the Tianjin Medical University General Hospital Ethical Committee. Three neuropathologists independently classified the histopathological diagnosis of glioma tissues according to the typical histopathological findings.

## Data Availability

All data generated or analyzed during this study are available from the corresponding author upon reasonable request.
